# Life-Cycle and Genome of OtV5, a Large DNA Virus of the Pelagic Marine Unicellular Green Alga *Ostreococcus tauri*

**DOI:** 10.1371/journal.pone.0002250

**Published:** 2008-05-28

**Authors:** Evelyne Derelle, Conchita Ferraz, Marie-Line Escande, Sophie Eychenié, Richard Cooke, Gwenaël Piganeau, Yves Desdevises, Laure Bellec, Hervé Moreau, Nigel Grimsley

**Affiliations:** 1 Université Pierre et Marie Curie-Paris 06, Laboratoire Arago, Banyuls-sur-Mer, France; 2 CNRS, UMR7628, Laboratoire Arago, Banyuls-sur-Mer, France; 3 Institut de Génétique Humaine, Génopole Montpellier Languedoc-Roussillon, UPR1142, Montpellier, France; 4 Génopole Languedoc-Roussillon, Génome et Développement de Plantes, UMR5096, Perpignan, France; University of Wyoming, United States of America

## Abstract

Large DNA viruses are ubiquitous, infecting diverse organisms ranging from algae to man, and have probably evolved from an ancient common ancestor. In aquatic environments, such algal viruses control blooms and shape the evolution of biodiversity in phytoplankton, but little is known about their biological functions. We show that *Ostreococcus tauri*, the smallest known marine photosynthetic eukaryote, whose genome is completely characterized, is a host for large DNA viruses, and present an analysis of the life-cycle and 186,234 bp long linear genome of OtV5. OtV5 is a lytic phycodnavirus which unexpectedly does not degrade its host chromosomes before the host cell bursts. Analysis of its complete genome sequence confirmed that it lacks expected site-specific endonucleases, and revealed the presence of 16 genes whose predicted functions are novel to this group of viruses. OtV5 carries at least one predicted gene whose protein closely resembles its host counterpart and several other host-like sequences, suggesting that horizontal gene transfers between host and viral genomes may occur frequently on an evolutionary scale. Fifty seven percent of the 268 predicted proteins present no similarities with any known protein in Genbank, underlining the wealth of undiscovered biological diversity present in oceanic viruses, which are estimated to harbour 200Mt of carbon.

## Introduction

Viruses are by far the most abundant biological entities in the oceans [1], [2], but relatively little is known about their life cycles and how they influence the diverse worldwide populations of plankton. Unicellular phytoplankton are at the base of the food web and recent evidence suggests that their populations are controlled to a large extent by viruses 2–7. Indeed, some authors suggest that viral infections drive diversity and speciation in the microbial world [8]. In order to gain some understanding of how such global ecosystems function, one important approach is to analyze in detail representative components of these interactions, and then propose hypotheses applicable to the behavior of the whole system. Historical experience with model systems shows that the choice of the interaction investigated is crucial, since our depth of understanding will be limited by the amenability of the partners in question to experimental analyses.

We focus this study on a virus of *Ostreococcus tauri*, the latter being the smallest eukaryotic free-living photosynthetic organism known, whose complete genome sequence has been analysed [9]. *O. tauri,* first isolated from the Thau Lagoon in 1994 [10], [11], is a free-living picoeukaryotic green alga (cell diameter ∼1 μm) with a minimal cellular structure and a relatively fast cell cycle in culture (2–3 divisions/day in continuous light). Its single chloroplast, single mitochondrion and cytoplasm are bounded by a membrane lacking any detectable cell wall or scales. In the marine environment, *Ostreococcus* sp have been found everywhere where suitable analytical techniques have been employed to detect them, such as in coastal and oligotrophic North Atlantic waters, in the Mediterranean, Indian and Pacific Oceans [12]–[14], and strains are available from many locations [15]. Analysis of seawater from many worldwide locations has shown that the genus *Ostreococcus* is common at depths of 0–120m below the surface where it contributes to varying extents to primary production [12], [16]. Different strains show specific adaptations to different environments (depth, light intensity, [17]) and the complete genome sequence analysis of 2 strains representative of such niches has been completed [9], [18], further strengthening the choice of this species as a model for host-virus interactions.

The presence of viruses in a coastal algal bloom of *O. tauri* has previously been observed [19], but these were not characterized further. We describe the isolation, growth in culture, life-cycle and gene content of *Ostreococcus tauri* virus 5 (OtV5), a DNA virus of OTH95, the best-characterized strain of *O. tauri* . We show by phylogenetic tree reconstruction with related viral taxa that OtV5 is a phycodnavirus, thus representing a commonly found group of marine virioplankton. Phycodnaviruses are nuclear-cytoplasmic large double-stranded DNA viruses (NCLDV) whose ancestors probably predate the separation of the eukaryotic kingdoms, with viruses that attack for example reptiles and mammals (such as Poxviruses) [20], [21]. Genomes of algal phycodnaviruses identified to date are 150 to 560kb in size [22] (http://www.ncbi.nlm.nih.gov/ICTVdb/Ictv/fs_phyco.htm). Viruses attacking the Prasinophyte *Micromonas pusilla* (previously known as *Chromulina pusilla* [23]), a common very small (1–3 μm) phytoplankter in coastal waters that often dominates in the “ultraplankton” [24], were first observed about 20 years ago[25], and the growth cycle of such a virus was subsequently observed in culture[26]. Since then, several authors have studied *Micromonas* viruses [27]–[32]. Important progress in studying the diversity of phycodnaviruses was enabled by the design of degenerate PCR primers using a conserved region of the polymerase gene of *Micromonas* viruses [33], since this sequence now serves as a phylogenetic landmark for both metagenomic data and newly characterized NCLDV sequences. Despite the global ecological importance of marine viruses suggested by metagenomic data [34] characterisation of phycodnavirus genomes has been limited until now to a few organisms including marine brown algae, *Ectocarpus siliculosus* [35], *Feldmania irregularis* (FirrV) [36] and *Emiliania huxleyi* [37], [38] , and to certain freshwater *Chorella* species, [39]–[41]. To our knowledge, we present the first whole genome analysis of a virus affecting marine green algae.

## Results

### Isolation and culture of OtV5

**Figure 1:**
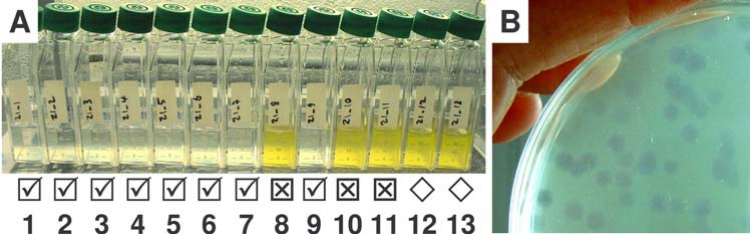
Assays for the presence of lytic viruses. A: Marine samples from Thau Lagoon (43° 24′N, 3° 36′E) incubated with the host strain OTH95 (isolated from the same place 14 years previously). Ticked boxes indicate lysis, crossed no lysis, and diamonds show mock-inoculated host cells. B: Viral lysis plaques in a plated gel of host cells.

The presence or absence of viruses in water samples was assayed by filtering the water to eliminate bacterial cells and protists, then mixing the filtrate with cultures of the host, *O. tauri*. Lysis of the host cells was usually observed about 1 week after inoculation (Figure 1A). By mixing a sufficient number of host cells (typically about 10^8^ cells) in culture medium with 1 or 10 ml of seawater before plating in the soft gel, individual plaques could be visualized in a growing green lawn on the plate (Figure 1B, see the materials and methods [M&M] section).

In a preliminary study carried out in 2006 (56 samples tested), viruses specific to the strain OTH95 (i.e. giving lysis plaques on this strain) were detected in 19 out of 37 samples from local saltwater lagoons while 2 out of 19 coastal seawater samples tested positive by lysis of OTH95.

OtV5 was chosen because it reliably gave a relatively rapid lysis of host cells (2–3 days to clearing of the lysate, depending on the inoculum density, and because it produced a relatively high titre of viable particles in suspensions (typically 10^8^ plaque forming units [pfu] in a cleared lysate), OtV5 was purified twice by single-plaque isolation before further studies.

### Host specificity of OtV5

Using a microtitre dish assay (M&M) the host range specificity of OtV5 was evaluated on a series of different picoplankton species, mostly isolated from the N.W. Mediterranean sea (Table 1). Neither more distantly related species (*Synechococcus* sp., *Tetraselmis* sp. *Dunaliella salina Nannochloris* sp., or *Pycnococcus* sp.,) nor more closely related Mamiellales species (*Bathycoccus* sp., or *Micromonas* sp.) were affected. More unexpectedly, among the 18 independently and locally isolated *Ostreococcus* spp. tested, none was affected, despite the fact that at least 9 of these strains had identical 18S rDNA sequences, the same as the host species OTH95 [17], [42]. OtV5 thus shows strict host strain specificity.

**Table 1:** Strain specificity of OtV5.

Species	Class	Clade [17], [42]	Strain^a^	Lysis by OtV5	Reference
*Ostreococcus tauri*	Prasinophyceae	C	RCC745	+	[10]
*Synechococcus sp*	Chroococcales			-	G. Vétion, pers. comm.
*Dunaliella salina*	Chlorophyceae			-	G. Vétion, pers. comm.
*Tetraselmis sp*	Prasinophyceae			-	G. Vétion, pers. comm.
*Bathycoccus prasinos*	Prasinophyceae	-	RCC1105	-	*
*Micromonas pusilla*	Prasinophyceae	A	RCC827	-	[15], [90]
*Nannochloris* sp.	Trebouxiophyceae	unkown	BCC42000	-	*
*Pycnococcus* sp.	Prasinophyceae	unkown	BCC41000	-	*
*Ostreococcus tauri*	Prasinophyceae	C	RCC1108	-	*
*Ostreococcus tauri*	Prasinophyceae	C	RCC1118	-	*
*Ostreococcus tauri*	Prasinophyceae	C	RCC1117	-	*
*Ostreococcus tauri*	Prasinophyceae	C	RCC1114	-	*
*Ostreococcus tauri*	Prasinophyceae	C	RCC1558	-	*
*Ostreococcus tauri*	Prasinophyceae	C	RCC1559	-	*
*Ostreococcus tauri*	Prasinophyceae	C	RCC1112	-	*
*Ostreococcus tauri*	Prasinophyceae	C	RCC1111	-	*
*Ostreococcus tauri*	Prasinophyceae	C	RCC1115	-	*
*Ostreococcus* sp.	Prasinophyceae	D	RCC1107	-	*
*Ostreococcus* sp.	Prasinophyceae	D	RCC1122	-	*
*Ostreococcus* sp.	Prasinophyceae	D	RCC1119	-	*
*Ostreococcus* sp.	Prasinophyceae	D	RCC1120	-	*
*Ostreococcus* sp.	Prasinophyceae	D	RCC1121	-	*
*Ostreococcus* sp.	Prasinophyceae	D	BCC15000	-	*
*Ostreococcus* sp.	Prasinophyceae	unkown	RCC1123	-	*
*Ostreococcus* sp.	Prasinophyceae	unkown	RCC1561	-	*
*Ostreococcus* sp.	Prasinophyceae	unkown	RCC1110	-	*

* strains isolated in the course of this work.

a RCC–Roscoff Culture Collection, BCC–Banyuls collection, cryopreserved.

### Life-cycle of OtV5

Routinely, inoculation of a late exponentially growing or stationary phase cultures (2×10^7^–8×10^7^ cells/ml) of host OTH95 cells with a fresh 0.2 μm-filtered OtV5 viral lysate (>10^8^ pfu/ml) cleared the host suspension within 48h. In order to characterize the kinetics of viral growth and development of OtV5 particles, this process was monitored by flow cytometry, electron microscopy, plate assays, and pulsed field gel electrophoresis (PFGE) of DNA from whole cells embedded in agarose at different stages of the infection.

**Figure 2:**
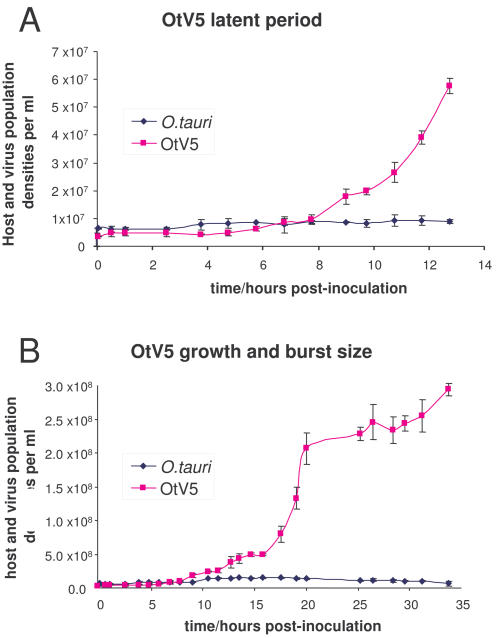
Latent period and burst size of OtV5. The numbers of viruses in suspension starts to increase 8 to 9 hours post-inoculation (panel A) and rises to a first plateau at 12 to 16 hpi (panel B), giving a burst size of 25 after correction for the number of infected cells (see “Results”). Thereafter, a further cycle of infection can be seen by the 2nd increase in OtV5 at 16-20hpi.

Firstly, we attempted to measure burst size and latent period using a moi (multiplicity of infection) of one. Cultures of exponentially growing host cells (6.4×10^6^±0.5×10^6^ cells/ml) were inoculated with a suspension of viral particles, estimated by flow cytometry to give 5.5×10^6^±0.9×10^6^ viruses/ml. By flow cytometry, we estimated that 36%±12% had adsorbed to the cells within 5 minutes, and that non-adsorbed viruses then remained in suspension when mixed with the cells, and appeared not to adhere to cells for at least several hours (Figure 2A). By serial dilutions and plating out a sample of the suspension from 5 minutes post-inoculation, we estimated that 2.1×10^6^±0.5×10^6^ cells/ml OTH95 host cells were actually infected. Since the number of viruses inoculated was 5.5×10^6^±0.9×10^6^ and 36% of them had adsorbed, we would expect to obtain about 2.0×10^6^ infected cells/ml. We thus conclude that all (or nearly all) of the adsorbed viruses were infectious. The number of viruses in the suspension started to increase appreciably after a latent period of 8 hours post-inoculation (hpi) , and the curve leveled out at 12 to 16 hpi at 5.1×10^7^±0.9×10^7^, giving a burst size of 25 viruses per cell. A further round of infection occurred after 16 hpi, leveling out after 20hpi (Figure 2B).

**Figure 3:**
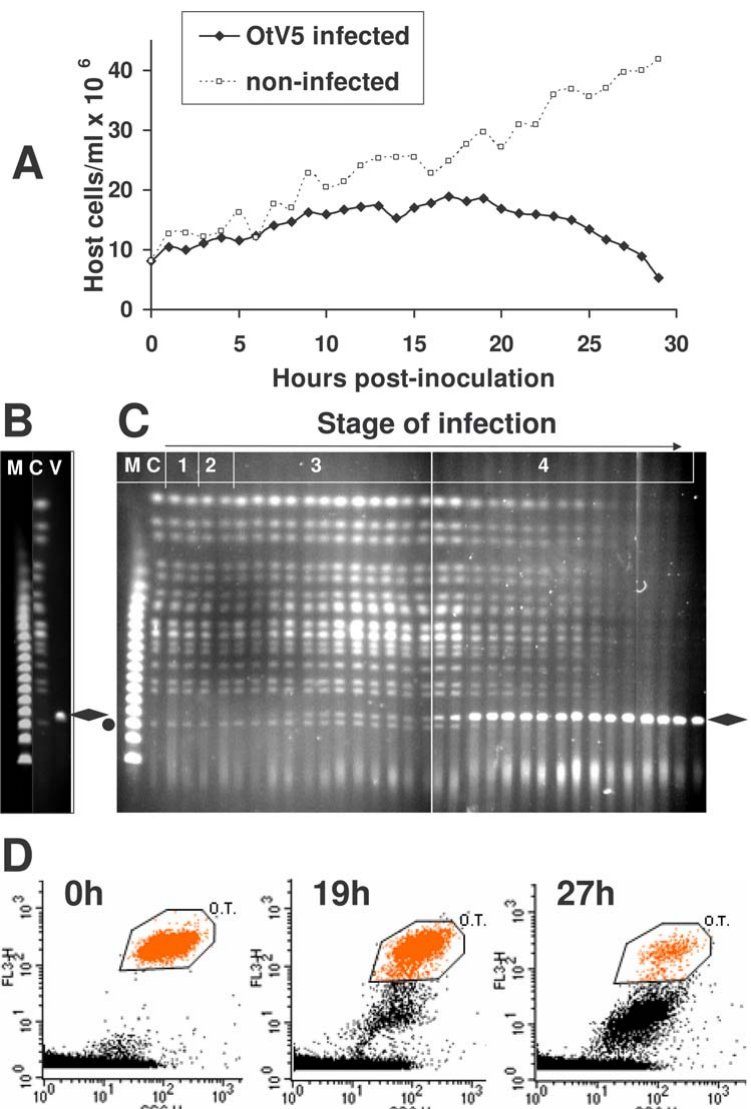
Time course of host cell lysis, karyotype, and production of OtV5 DNA during infection. A: host cell density in a control culture (open symbols) and a culture inoculated with OtV5 (filled symbols). B: Pulsed Field Gel Electrophoresis of samples from control DNAs and C: following the time course shown in A, showing *O. tauri* chromosomes and viral DNA. M–marker concatenated bacteriophage λ DNA ladder, C–control non-inoculated cultures, V–isolated OtV5 DNA. Stage 1–a culture 30sec pre- and 30sec post-inoculation, stage 2–20min and 40min post-inoculation, stages 3 and 4–at 60min and 1h intervals thereafter (images from 2 gels were pasted together to accommodate the number of gel tracks necessary). The smallest host chromosome migrates at the position of the 145kb marker band (dot), and the virus-specific DNA band migrates around 180kb (diamond). D: Flow cytometric enumeration of *O. tauri* analysis using autofluorescence of chlorophyll a as a marker. The window “O.T.” contains cells showing autofluorescence typical of healthy growing cells. At 19 hours after infection, about 20% of the cells have died as shown by reduced fluorescence (ordinate axis). At 27h about 90% have died and about 1% remains at 43h.

**Figure 4:**
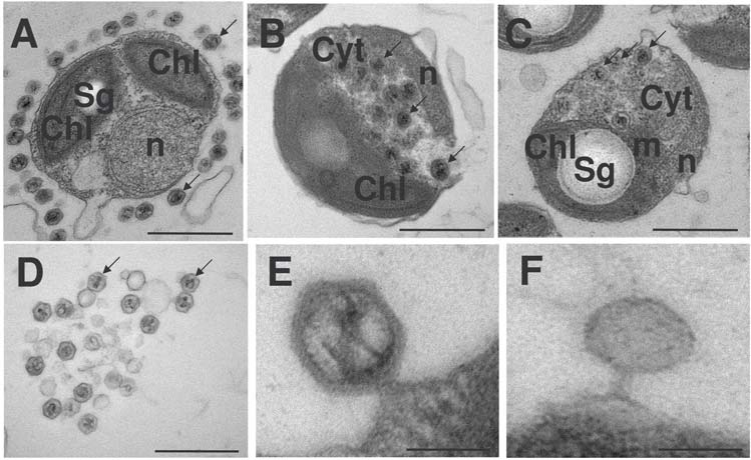
Electronic microscopy of infected *Ostreococcus tauri* cells. A, B, C, D, the bar represents 500nm; E, F, bar 50nm. Example virus particles are shown with arrows. Chl–chloroplast; Cyt–cytoplasm, n–nucleus, m–mitochondrion, sg–starch grain. A–at high moi many viruses can adsorb to a single cell. In B, C, D, E, & F the moi was one. B & C show viruses accumulating in the cytoplasm before cell lysis occurs. D–virus particles clumped together around a lysed cell, E (30min after infection)–full virus particle attached to plasmalemma, F–empty particle.

Secondly, we verified that the OtV5 genome could be prepared and visualized on PFGE gels (Figure 3B, band migrating around 180kb, see M&M). This band was RNAse-insensitive and disappeared after treatment with DNAse (data not shown). Thirdly, a one liter culture of the host OTH95 strain in late exponential growth (10^7^cells/ml) was inoculated with a fresh lysate of OtV5 at a moi of 1 pfu per host cell. We show (Figure 3A) that after inoculation the culture continued to grow for 8h, albeit more slowly than the mock-inoculated control flask, attaining a plateau around 1.5×10^7^cells/ml for 10–12h, before a progressive decrease in the number of autofluorescing cells over the next 12h period. PFGE analysis at frequent intervals during the time course permitted visualization of the genomes of both the host and the virus. At early stages of the infection, the host chromosomes can be seen, but no viral genomic DNA is visible (Figure 3C early stages 1 & 2). Full-length viral genomes appear about 2 hpi (hours post-inoculation, see Figure 3C. mid-late stage 3), as seen by the ∼180kb band migrating close to the smallest chromosome of *O. tauri*, showing that the viral DNA replicates rapidly in host cells in this period. After 8h the overall number of copies of viral genomes visible increases dramatically as might be expected since newly produced viruses are released into the medium at this stage (Figure 2B); a large proportion of them probably adhere quickly to cells in the culture (see below, Figure 4A), and would thus be visible by PFGE after harvesting the host cells by centrifugation. After 30 hpi numbers of autofluorescing host cells decrease considerably. Remarkably, the host cell chromosomes remain intact throughout this entire period. Thereafter the diminishing quantities of host chromosomal DNAs reflect the decrease in the number of cells showing a level of autofluorescence typical of healthy cells.

### Electron microscopy

Compared to the size of OTH95 cells (about 1 μm), the icosahedral particles of OtV5 were relatively large (122±9)nm, complete particles containing a central region of unevenly electron-dense material (see Figure 4E, for example). Using a high moi (2000 viruses per host cell in suspension), we observed that many viruses could adsorb to a single cell within 5 minutes of inoculation (Figure 4A). It is not clear whether the viruses first adhere to an electron-transparent layer surrounding the cell membrane, or whether they form a “bridge” directly with the host cell that lies outside the thin section. About 20% of sections from healthy cells (48/218 cells) appeared to have viruses adsorbed, whereas no viruses were visible on the sections of the remaining host cells, despite the very high moi used (plate 4A). We did not see any “tail” on the virus particle, but OtV5 could be seen attaching by a vertex to the external membrane of OTH95 by a bridge between the cytoplasm and the virus. Using an moi of 1, apparently complete virus particles appeared in the cytoplasm at 6 hpi (Figure 4 B). Viral particles were localized to a limited part of the cytoplasm. At 14 hpi 18% (42/229 cells) of mid-cell TEM sections showed particles. No viruses were found in the nucleus, the chloroplast or mitochondria. They usually occupied a space near the inner face of the nucleus, between it and the mitochondria or the chloroplast.

### Characterisation of the viral genome

**Figure 5:**
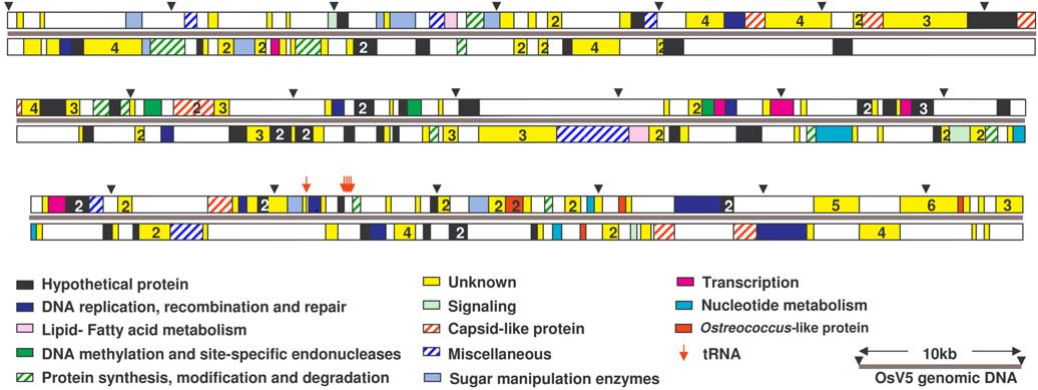
Features of the OtV5 virus genome. Predicted coding sequences are represented by colored rectangles (white–the reading frame is on the opposite DNA strand). The bar and filled triangles indicate map lengths in kb.

Nucleic acid was isolated using standard techniques for manipulation of high M.W. DNA (see M&M). Briefly, a 2l culture of a fresh lysate was concentrated by ultrafiltration, and the concentrate incorporated into an agarose plug, and run out on a PFGE preparative gel before recovery of the viral genomic DNA band. This band was RNAse-insensitive (data not shown) and was used for preparation of a shotgun library. The genome is 186,234 base pairs long (Figure 5), in agreement with the size expected from migration of dsDNA by PFGE (Figure 3B and C). Its GC content (45%) is lower than that of the host (58%, [9], see Table 2). Terminal inverted repeats (TIR) of 1695 bp are predicted, their boundaries being defined by contigs spanning both the IR and flanking genomic DNA which were clearly identified in the assembly of the genome sequence. In addition, we showed that the left or right TIR could be amplified independently by PCR using primers in the adjacent single copy DNA (data not shown).

**Table 2:** Comparison of the general characteristics of the genomes of OtV5 with *Ostreococcus tauri* strain OTH95 and other algal viral genomes.

	OtV5	*^2^O. tauri* [9]	PBCV-1 [91]	Ehv-86 [37]
Genome size (bp)	186,234	12,560,000	330,743	407,339
% G+C	45	58	40	40
Predicted CDSs (≥65 aa)	268	8,166^b^	367	472
Mean gene size (bp/gene)	692	1300	899	863
% known genes	22	} 77	} 50	} 21
% hypothetical genes	20			
% genes with no hit^a^	57	23	50	79

a BLASTP E-value threshold 10^−5^

b includes all predicted CDSs

### Genome annotation

**Figure 6:**
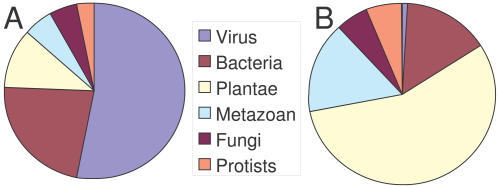
Taxon distribution of best BLASTP hits. A: using OtV5 viral genes. B: using host *Ostreococcus tauri* genes.

Seven hundred and thirty six ORFs (open reading frames) ≥65 amino acids (aa) long were considered, but 468 were discarded either because they did not possess an N-terminal methionine or because they appeared within other ORFs. Two hundred and sixty eight CDSs (coding sequences, defined here as ORFs flanked by start and stop codons) remained, thus giving a similar gene density to that of PCBV-1 (1.11 CDS/kb for PCBV1 and 1.45 for OtV5). Eighteen per cent of predicted CDSs overlap, but this is not unexpected because some viruses are known to use polycistronic mRNAs [43]. Conceptual translations of a high proportion (57.5%) of the predicted CDSs showed no similarity to proteins with known functions using BLASTP alignment with public databases [44] (Figure 6). Genes of different functional categories and the transcriptional direction of these predicted CDSs are apparently distributed randomly on both DNA strands through the genome. Proteome analysis using Pfam identified many possible functional domains (Table S1). Five closely clustered tRNA-encoding sequences were found, including 2 with introns (see Genbank accession number EU304328).

**Figure 7:**
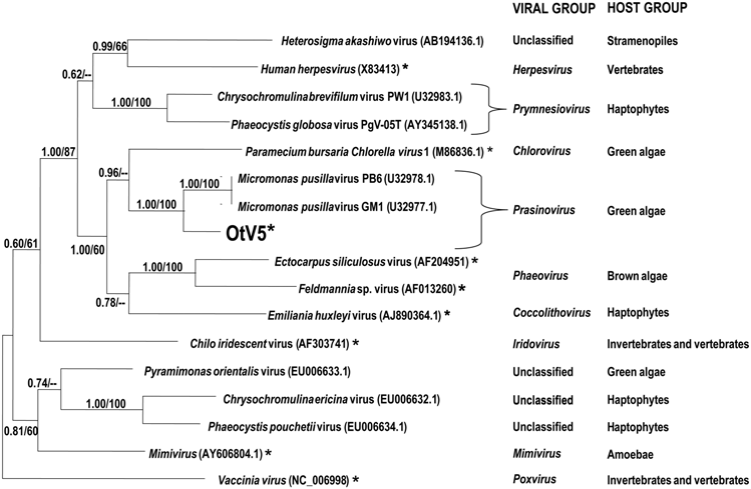
Phylogenetic tree showing OtV5. Asterisks indicate that at least one complete virus genome is available within the group. A phylogenetic tree computed by Bayesian analysis (BI) and maximum likelihood (ML) analyses on the partial DNA polymerase gene DNA sequences of selected taxa of the family Phycodnaviridae and other large dsDNA viruses. Genbank accession numbers of reference taxa are given in parentheses. The position of OtV5 is indicated in large bold type. Numbers are posterior probabilities (BI) and bootstrap proportions (ML; values below 50 are indicated by “–”) reflecting clade support.

Many enzymes potentially involved in the replication of viral DNA are predicted (Table 3, and EU304328). The predicted DNA polymerase, which is used as a reference for classification of large dsDNA viruses, shows greatest similarity to those of *Micromonas* viruses. A phylogenetic tree was made by comparing a conserved part of this gene with homologous sequences from other nuclear cytoplasmic large DNA viruses (NCLDV, [45]), with emphasis on algal DNA viruses (Figure 7). These observations, together with the presence of 31 genes whose predicted functions are conserved in other algal viruses (see below and GenBank accession number EU304328), including “core genes” of NCLDV [45], and our observations of the DNA genome size and particle morphology, support the inclusion of this virus in the phycodnavirus group [46], [47].

**Table 3:** Similarities to OtV5 predicted proteins

CDS#	Gene names	BBH	GG	CDS#	Gene names	BBH	GG
	**DNA, RNA, replication, recombination and repair**				**Lipid–Fatty acid metabolism**		-
240f	DNA polymerase	V	**+**	040f	Oxo-acyl (acyl-carrier protein) dehydrogenase	B	**-**
205r	ATP dependant DNA ligase	O	**-**	136r	Patatin-like phospholipase	V	**+**
199f	DNA topoisomerase	B	**+**		**Protein synthesis, modification and degradation**		
244r	DNA topoisomerase	V	**+**	092f	N-myristoyltransferase	M	**-**
115f	PCNA	V	**+**	126r	Prolyl 4-hydroxylase	V	**+**
067f	Helicase	V	**+**	152r	33 kDa in vitro translation peptide	V	**-**
005r	Exonuclease	B	**+**	171r	33 kDa in vitro translation peptide	V	**-**
146f	Exonuclease	V	**+**	013r	FtsH2 (3-4-24) metalloendopeptidase	B	**-**
098r	ATPase (DNA packaging)	V	**+**	042f	Aminotransferase family protein	M	**-**
194f	Rnase H	B	**+**	028r	Asparagine synthase	B	**-**
	**DNA methylation and site-specific endonucleases**			041r	NAD-dependant epimerase dehydratase	B	**-**
097f	Adenine DNA methyltransferase	B	**-**	203f	Aspartyl/Asparaginyl beta-hydrolase	B	**-**
144f	Adenine DNA methyltransferase	V	**-**	226f	Aspartyl/Asparaginyl beta-hydrolase	B	**-**
124f	M6A-gamma-methyltransferase	V	**-**	094f	Zinc metallo protease	V	**+**
	**Sugar manipulation enzymes**				**Signaling**		
011f	GDP-D-mannose dehydratase	M	**-**	030f	Potassium channel protein	B	**+**
012r	Glycosyl transferase	B	**+**	237r	Rhodanese-like domain protein	B	**+**
020r	Glycosyl transferase	V	**+**	168r	Serine/Threonine protein kinase	B	**+**
035f	Glycosyl transferase	B	**+**		**Capsid proteins**		
037f	Acetolactate synthase (2-2-1-6)	B	**-**	068f	Capsid protein 1	V	**+**
197f	6-Phosphofructokinase	B	**-**	076f	Capsid protein 2	V	**+**
043f	dTDP-D-glucose 4,6-dehydratase	M	**-**	081f	Capsid protein 3	V	**+**
218f	dTDP-D-glucose 4,6-dehydratase	M	**-**	099f	Capsid protein 4	V	**+**
	**Transcription**			100f	Capsid protein 5	V	**+**
177f	Transcription factor IIB	B	**+**	190f	Capsid protein 6	V	**+**
024r	Transcription factor IIS	V	**+**	239r	Capsid protein 7	V	**+**
149f	Transcription activator	V	**+**	243r	Capsid protein 8	V	**+**
161f	Tata-box Binding Protein (TBP)	V	**-**		**Miscellaneous**		
145f	RNase III	V	**+**	039f	3-Dehydroquinate synthase	B	**-**
	**Nucleotide metabolism**			188r	ATP/GTP binding site motif A AGB-1	V	**+**
153r	Ribonucleotide reductase large subunit	B	**+**	O14f	Methyltransferase FkbM	B	**-**
174r	Ribonucleotide reductase small subunit	M	**+**	135r	Viral A-type inclusion protein	M	**-**
227r	Thymidine kinase	G	**+**	059f	Flavin-dependant thymidylate synthase	M	**-**
231f	dUTP pyrophosphatase	V	**+**	180f	mRNA capping enzyme	V	**-**

BBH–best BLASTP hit similar to: B–Bacteria, G–Viridiplantae (green plant lineage), M–Metazoa, V–Virus. GG–predicted gene present in other viruses of Chlorophyta (Green Gene)

**Figure 8:**
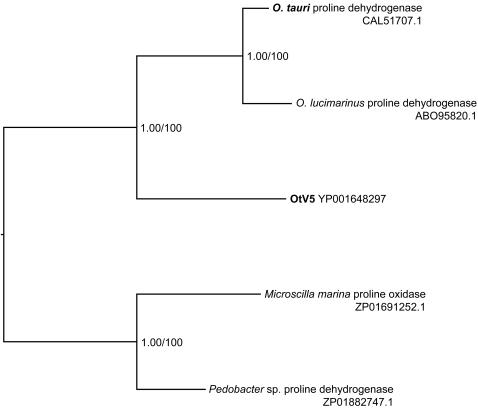
Phylogenetic tree of OtV5 proline dehydrogenase. A phylogenetic tree computed by Bayesian analysis (BI) and maximum likelihood (ML) analyses on partial Proline dehydrogenase and Proline oxidase aa sequences. Genbank accession numbers of taxa are given in parentheses. The position of OtV5 and *O. tauri* are indicated in bold type. Numbers are posterior probabilities (BI) and bootstrap proportions (ML).

Notably, several host-like DNA sequences (HLS) were identified in the viral genome. Six potential CDSs whose conceptual translations show significant similarity to *Ostreococcus spp*. genes were identified by their high BLASTP scores. Phylogenetic analysis of these 6 candidates (data not shown) grouped 4 ORF with other viral proteins, thus excluding the likelihood of recent horizontal transfer, and 2 other ORF together with eukaryotic sequences. However, the viral and host sequences are too divergent to make definitive conclusions about their evolutionary origins. Additional viral and host sequences from a range related taxa are needed to clarify this point. The largest of the HLS (253 aa), for which we present a phylogenetic comparison (Figure 8) encodes a potentially complete proline dehydrogenase gene.

## Discussion

Viruses infecting *O. tauri* could be found frequently in water samples collected from marine lagoons and open sea in the N. W. Mediterranean. They were found about five times more frequently in lagoons than in the sea (51% of samples vs. 11% of samples, respectively), perhaps reflecting the preferred habitat of its clade C [17], [42] host. Detailed ecological observations and analysis of their phylogenies will be published elsewhere. Here we focus our attention on one virus, OtV5, isolated from the Bages lagoon on 24^th^ January 2006, which lysed host cultures reproducibly and rapidly (within 2 days, Figure 2 and 3). We tested the strain specificity of OtV5 on diverse picoeukaryotic strains of algae that were isolated and cloned mostly from local seawater samples between January 2006 and March 2007. Firstly, using 5 different genera of Prasinophyceae or 2 different clades of *Ostreococcus spp*., no strains were susceptible. Secondly, none of a set of 9 independent and genetically polymorphic clade C strains was attacked by OtV5, which thus shows very narrow strain specificity. While further studies with larger sets of hosts and strains are required but are beyond the scope of this study, we note that this situation may resemble the gene-for-gene resistance arms race observed in higher plant-pathogen interactions [48], [49], where natural selection drives diversity in plant disease resistance genes. Relatively little is known about the selective forces driving the diversity and speciation of microbes in the marine environment, but viruses appear to play a key role in these processes that furnish the base of the global food web [5], [7], [8], [32], [50]–[55].

The extremely small *O. tauri* cell is crowded with organelles, which are often observed to deform the apposed plasmalemma, and the cytoplasmic compartment represents a maximum of about 30% of the *O. tauri* cell volume [56]. If all of this were packed with viruses, about 100 particles might be accommodated. However this volume also accommodates vesicles and hundreds of ribosomes, virus particles being localized to a limited part of the cytoplasm. It is thus not surprising that the mean burst size is only 25 particles per cell. EM pictures showed a maximum of 15 particles in a 50nm thick section of a single cell. These data are comparable to the burst size of 70–100 particles per cell observed for the related host/virus system *Micromonas pusilla/MpV* [26], considering the host cell volume of *Micromonas* is about 4 times more voluminous than *Ostreococcus* (the diameter of *O. tauri* in our culture conditions is about 1 μm [Figure 4] and *Micromonas* is 1.6 μm [57]).

Using a high moi, EM also showed that many virus particles could adsorb to a single cell (Figure 4A) but that high numbers of viruses apparently adsorbed to only a proportion of the cells (about 20%). This observation was confirmed by flow cytometry at lower moi (at a moi of one, about 35% of viruses were adsorbed). The remaining majority of particles then remained in suspension following inoculation despite the presence of an actively growing population of host cells (Figure 2A), suggesting that such particles lacked a host cell attachment function, and/or that the remaining cells were resistant to infection. The latter hypothesis, however, seems unlikely given the clonal nature of the host strain. The above information, together with the actual number of infected host cells estimated by pfu (see the “Results” section), suggests that viral attachment may be a limiting step in the infection. At high moi 5 min after inoculation, a clear layer about 50nm thick separated the particles from the cytoplasm (Figure 4A). Since apparently few of the viruses have formed a “bridge” with the cytoplasm at this stage (the EM slice is 50nm thick), we favor the hypothesis that the cell is protected by a cellular envelope, never previously observed in this algal genus, that is invisible in our EM preparations. The biochemical nature of this putative envelope remains to be determined.

We could not however use EM images from high moi to determine when the particles “injected” their electron-dense contents, because all of the cells with a large numbers of viruses attached deteriorated rapidly (<20min, not shown) and disappeared from the culture, perhaps due to multiple perforations of the membrane or to degradation caused by injection of unknown virion proteins, involved in virulence, as apparently may occur in related NCLDV [58]. Host cells with “empty” virus particles attached (Figure 4F) were rarely seen at any stage of the infection, suggesting that they might usually detach from the cells after injecting their contents.

The average size of predicted CDSs in the linear double-stranded 186,234 bp DNA genome is similar to other phycodnaviruses. One hundred and fourteen CDSs (42.5%) encoded predicted proteins with similarities to other hypothetical proteins in databases, but only 60 of these (22.4%) showed similarity with proteins to which a function has been attributed. This last category may, however, be overestimated as some of these may not be bona fide CDSs. Fifty four (20.1%) CDSs matched other hypothetical CDSs of unknown function, of which 28 (10.5%) came from other viruses, and 26 (9.7%) from elsewhere, and 154 (57.5%) of genes were unknown, Finally, predicted products of 154 genes (57.5%) correspond to no known protein, emphasizing the extremely diverse range of uncharacterized biological functions potentially represented in the phycodnaviruses.

In common with other algal viruses (Table S2), numerous enzymes involved in DNA replication and gene expression are predicted. This is an ancient common feature of the super-group of NCLDV, including phycodnaviruses, which retain a set of predicted functions that presumably assure their life-cycles in the host cytoplasm. Like PBVC-1, the OtV5 genome shows terminal inverted repeat sequences, and its ends are thus likely to form hairpin loops [59]–[61]. Our phylogenetic reconstruction shows that OtV5 clearly clusters with phycodnaviruses infecting the prasinophyte *Micromonas pusilla* [33]. These latter viruses form the Prasinovirus clade within Phycodnaviridae, and Prasinovirus should then include OtV5, from a prasinophyte host. Our tree is different from that of Schroeder et al. (2002), because we use different methods for the analysis, giving higher node support values (posterior probabilities). However, in both trees, Phycodnaviridae (Phaeovirus+Coccolithovirus+Prasinovirus+Chlorovirus+Prymnesiovirus) do not form a monophyletic group, with Herpesviridae and *Heterosigma akashiwo* virus nested inside (Figure 7). NCLDV, which attack hosts as diverse as man, reptiles, fishes, invertebrates and algae, are thought to have evolved from a common ancestor, with a core set of common genes [20], [45]. Like some of its poxviral cousins [62], OtV5 probably accumulates embedded in a cytoplasmic region rich in a viral-A type inclusion protein, since a CDS whose product shows similarity to this protein was identified (Table 3 and Genbank accession number EU304328).

We found no evidence of site-specific endonucleases which are present or predicted in some other previously characterized phycodnaviruses[35], [37], [63]–[65], although similarities to 3 different methylation/modification enzymes are present. DNA degradation in host *Chlorella* cells by PBCV-1 (*Paramecium bursaria Chlorella* virus 1)is an early viral function, occurring within 5 minutes of host cell penetration [65], in stark contrast to OtV5, where host chromosomes remain intact throughout the viral life-cycle (Figure 3C). In this respect, OtV5 resembles MT325, which also lacks predicted endonucleases [65]. However, in contrast to MT325, where complete viral genomes are present immediately after inoculation, OtV5 genomic DNA could not be visualized by PFGE within the first 2h after inoculation. Since flow cytometrical observations and pfu measurements show that about 35% of the cells have adhered viruses at this stage, we conclude that this level is below the level of detection by PFGE, and that viral genomes become visible after their replication inside host cells.

OtV5 encodes 3 predicted adenine methyltransferases that are possibly involved in protection of the host and/or the viral DNA, for example from the kind of programmed cell death response observed in other green algae [66], or from attack by a third party invader. Like its larger mimivirus relative [64], OtV5 encodes an asparagine synthase (AS) gene, and several viral tRNAs are predicted, including Asn-tRNA. In green plants, AS is a cornerstone of light-regulated protein synthesis, controlling the ratio of carbon and nitrogen anabolites available for protein or sugar syntheses diurnally [67]. OtV5 carries a CDS with similarity to FtsH metalloendopeptidase that is absent in other characterized eukaryotic algal genomes. Perhaps this enzyme performs a similar function to its homologue found in photosynthetic bacteria and plants, where it is involved in the repair maintenance of photosystem II [68], since the chloroplast remains intact throughout the viral life-cycle, and most likely functional as the starch grain continues to enlarge. However, all of the above hypotheses remain speculative and must await experimental investigation.

Interestingly, we found some indications for the capture of host DNA within the viral genome, since 6 predicted host-like sequences (HLS) present similarity to host genes (GenBank accession number EU304328). These are unlikely to be very recent transfers, because their GC content is clearly virus-like, (45%) rather than host-like (58%). Nevertheless they may represent an important evolutionary possibility for transfer of genetic information between host cells, since the amino acid sequences gave very high BLASTP scores with *Ostreococcus* spp. proteins, and have thus not had time to degenerate extensively by mutations, deletions, or other rearrangements. Remarkably, one of these CDSs encodes an apparently complete proline dehydrogenase gene. This enzyme is important for proline catabolism in all cellular organisms, is known to play a role in stress response in plants and in some other algae [69], and is used as a source of nutrients by numerous bacterial symbionts and pathogens [70], [71]; it could thus play an important role in some aspect of the host-virus interaction. Phylogenetic analysis suggests that the OtV5 gene could originate from bacteria (Figure 8). The AT content of this CDS is 55% in OtV5 *vs*. 41% in *O. tauri,* a highly significantly difference (p<10^−16^ [exact binomial test]). This difference is particularly striking on synonymous positions, suggesting that the functionality of this predicted protein was maintained by natural selection following its transfer to the virus, but a difference nevertheless remains on non-synonymous positions once the synonymous changes are removed (4-fold degenerate codons, p<10^−5^). The latter may arise from selective pressures for reducing the cost of viral genome replication and/or requirements for viral transcription and translation [72]. We have not found this predicted gene in other viruses in databanks, but in plants proline levels increase in stress conditions such as pathogen attack, and are controlled by proline degradation, inviting speculation that this enzyme plays a role in regulating the host stress response [69]. The CDS of the other HLS are smaller, but they nevertheless support the notion that exchange of sequences between host and viral genomes may be an evolutionarily frequent event. However, weak similarities between genes make it difficult to prove this with phylogenetic analyses, because of the absence of information from sufficiently closely related organisms.

The very small size of the cells and genomes of the *Mamiellaceae,* together with their worldwide distribution and their ecological importance, promise that they will provide key models for interdisciplinary approaches to global ecology. Several different complete Prasinophyte genomes have already been sequenced or are in the process of being analyzed, incuding 3 *Ostreococcus* spp. [9], [18], 1 *Bathycoccus* sp. (unpublished data) and 2 *Micromonas* spp. (A. Worden, personal communication). Viruses play a crucial role in the regulation of phytoplankton populations [5], [34], [52], [54], [55], [73], and genomic analyses should provide insight about the biological phenomena driving selection and diversity at the base of the food web.

## Materials and Methods

### Culture of host algal strains and virus OtV5

The host strain OTTH0595 was used for *Ostreococcus tauri* culture in all experiments, and cytometric observations made as previously described [10]. Cultures were grown in Keller-medium (Sigma-Aldrich, Saint-Quentin Fallavier, France) diluted in 0.22 μm filtered sea water (NaCl 36 g.L^−1^) under continuous light (100 μmol photon m^−2^s^−1^) at 20±1°C and under mild agitation. OtV5 was first identified as a single lysis plaque by inoculation of a sample of 1ml of 0.2 μm-filtered seawater taken from Bages Lagoon on 24/1/2006 into 20ml of a 1-week old host OTH95 culture plated at 1×10^7^ cells.ml^−1^ in 0.15% agarose. After two rounds of single-plaque purifications, viruses were subsequently produced by infecting liquid cultures of *O. tauri* either in exponential phase (e.g. 1×10^7^ cells/ml) or in stationary phase (about 8×10^7^ cells/ml). For preparation of large quantities of viruses for genome sequencing, two litres of an *O. tauri* exponentially growing culture (approx. 5.10^7^ cells ml^−1^) was inoculated with 200 ml of OtV5 lysate (approx. 5×10^8^ pfu ml^−1^, ratio *O. tauri*/OtV5 around 1). Lysed cultures were passed sequentially through a 5 μm and 0.45 μm filters to remove large cellular debris. Virus filtrates were concentrated by ultrafiltration with a 50K MW size cut-off unit (Millipore, Amicon Ultra) to a final volume of 6 ml. Virus concentrates were embedded in agarose for PFGE (see below). The density of virus particles in suspension was also estimated by flow cytometry [74]

### Plaque visualization assay

To facilitate the isolation and purification of viruses it was desirable to develop a plating technique in order to visualize individual lysis plaques. Since we were unsuccessful in growing the host cells on the surface of agar plates, we used a plate-gel technique for plating *O. tauri* embedded in a soft gel (N. West, personal communication). Lysis “plaques” could then be visualized as cleared circular regions extending through the whole depth of the gel. 3ml of an ∼3×10^7^ cells/ml growing culture of *O. tauri* were mixed with the viral inoculum in 0.1–10ml seawater and the volume brought to 20ml by adding K-medium and kept at 20° on the bench for ≥30min. Separately, a suspension of 1.5% agarose (Euromedex type D-5, DNA grade) was dissolved in distilled water by heating to 100°C, and then held at 65° in a water bath in tubes in 2.2ml aliquots before use. For plating, the 20ml of cell suspension was added to 2.2ml of hot agarose and mixed rapidly before pouring into a 9cm diameter Petri dish. The plates were cultured (continuous light 100 μmol photon m^−2^s^−1^, at 20±1°C) inside a transparent plastic box to maintain humidity for up to 1 to 2 weeks. Plaques could usually be visualised 3 to 8 days after inoculation as cleared areas on a green background. The titre of viable viruses present in the suspensions used for inoculations (and moi calculations) were measured routinely by counting lysis plaques in 2 parallel independent dilution series' on such OTH95 host plates

#### Flow cytometry

Analyses were performed on a FACScan flow cytometer (Becton Dickinson, San Jose, CA, USA) equipped with an air-cooled argon laser (488 nm, 15 mW). *Ostreococcus tauri* cells were characterized according to their right-angle scatter and their red fluorescence emission due to the chla pigment [75]. OtV5 viruses were determined both by their right-angle scatter and their fluorescence after SYBR green I staining. Viruses were stained according to [74].

#### Electron microscopy

For transmission electron microscopy (TEM), the cells were prepared according to Chretiennot-Dinet, 1995. They were fixed with glutaraldehyde in their culture medium at a final concentration of 1% and were concentrated by centrifugation for 15 min at 3000g. The centrifuged material was then mixed rapidly with 40 μl of 1% liquid agarose (agarose type II, Sigma) at 37°C using a microrepetor (SMI, Emerville, CA, USA). Once the agarose in the disposable glass micropipette had solidified, a “noodle” containing the cells was obtained and treated as a piece of agar, fixed for 2 h at 4°C in 2.5% glutaraldehyde with one volume of 0.4M cacodylate buffer and two volumes of culture medium (A solution). The noodle was then washed 3 times for 30 min in a buffer medium made of one volume 0.4M cacodylate buffer and one volume culture medium (B solution). Postfixation was carried out in 1% OsO_4_ in 0.2M cacodylate at 4°C for 1h. After two washes in 0.2M cacodylate, the noodle was cut in small pieces, dehydrated in a series of ethyl alcohol and embedded in Epon 812. Thin sections were stained with uranyl acetate and lead citrate before examination on a 7500 Hitachi transmission electron microscope.

#### Pulsed field gel electrophoresis (PFGE)

Agarose embedded *O. tauri* cells with or without OtV5 were analysed by PFGE as described previously [76]. From 1.10^7^ to 2.10^7^ cells were loaded per lane in a 1% agarose gel (Type D-5, Euromedex France) and the electrophoresis parameters were 6 V cm^−1^, 0.5× TBE, 120° switching angle, 14°C, 60 s switch time for 15 h then 90 s switch time for 9 h. The gel was then stained with ethidium bromide in order to visualise the chromosomal bands.

### OtV5 genome

Individual lots of about 1 μg of OtV5 nucleic acids were digested overnight at 37°C by DNase, or RNase (Sigma, France), or different restriction enzymes (HindIII, BamHI and SmaI; Promega France) and the digested products were analyzed by electrophoresis (1% agarose, 100V/cm, 20 min.).

Genomic DNA for sequence analysis was prepared by embedding viral particles in agarose strings at approximately 10^11^ particles/ml and lysing of the particles by proteinase K. Agarose was then digested by gelase (Epicentre, Tebu) and DNA was precipitated by ethanol. Purified DNA was broken by sonication, DNA fragments ranging from 1 to 5 Kb were separated in an agarose gel before purification and end-filling. Blunt-end fragments were inserted into pBluescript IIKS (Stratagene) digested by EcoRV and dephosphorylated. Plasmid DNA from recombinant *E. coli* strains was extracted according to the TempliPhi method (GE Healthcare) and inserts were sequenced on both strands using universal forward and reverse M13 primers and the ET DYEnamic terminator kit (GE Healthcare). Sequences were obtained with MegaBace 1000 automated sequencers (GE Healthcare). Data were analyzed, and contigs were assembled by using Phred-Phrap [77] and Consed software packages (http://www.genome.washington.edu). To collate data and facilitate annotation 2861 reads (each ∼600bp long) of DNA sequences derived from the shotgun library were assembled, giving 9.2-fold coverage. Remaining gaps (72) were filled by primer-directed sequencing on complete viral genome DNA using custom-made primers using a BigDye3 dye terminator sequencing kit (Applied Biosystems, Courtaboeuf, France) and analysed on an Applied Biosystems 3100 sequencer.

### Sequence annotation

The complete genomic sequence was annotated using the “Artemis” software [78], [79]. Amino acid sequences corresponding to all predicted CDS were screened against the curated Pfam-A profiles [80] for functional motifs using the perl script Pfam_scan.pl (Wellcome Trust Sanger Institute, UK) using default settings. The e-value cut-off of 1 was conserved as profiles were mostly defined from distantly-related sequences and many functional motifs with relatively high e-values are confirmed by significant BLASTP alignments [81] with proteins having the same putative function.

### Sequence retrieval and alignment

Reference partial DNA-polymerase DNA sequences from Phycodnaviridae and phylogenetically related large dsDNA viruses were gathered from Genbank. We chose reference taxa among those previously used for comparing algal viruses [82], then added Mimivirus [83] and new Phycodnaviruses relased in Genbank after this study. The homologous partial DNA polymerase sequence from OtV5 was aligned with these reference sequences, and the sequence of *Vaccinia virus* (Poxvirus) was used as outgroup following Lakshminarayan *et al.* (2006). Alignment was performed with ClustalX [84] and improved by eye with Se-Al v2.0a11 (Rambaut, A. 1996. Se-Al: Sequence Alignment Editor. Available at http://evolve.zoo.ox.ac.uk/). Ambiguous regions were deleted from the alignment before phylogenetic analysis, for a final length of 678 bp.

### Phylogenetic reconstruction

A phylogenetic tree including OtV5 was constructed by using the partial DNA polymerase sequence routinely amplified in other related viruses [33] by Bayesian analysis and Maximum likelihood. Bayesian analysis was done with MrBayes 3.1.2 [85]. The reconstruction used 4 chains of 10^6^ generations with an evolutionary model designed for coding sequences and considering each codon position as a distinct partition. Burnin value was set to 20% of the sampled trees (1% of the number of generations). Maximum likelihood was performed with PhyML [86], [87], using a General Time Reversible evolutionary model accounting for substitution rate heterogeneity and a proportion of invariable sites, chosen with ModelTest [88], and validated via a bootstrap procedure with 100 replicates. To screen the OtV5 genome for potential horizontal gene transfer candidates, we systematically screened OtV5 ORFs (>40 aa) for homology with the *O. tauri* genome using tblastn and retrieved 17 OtV5 ORFs with aa identity greater than 45% over more than 50 aa. We then searched for GenBank BBHs (Best Blast Hits, using blastp), and identified 6 ORFs that contain *O. tauri* sequences in the 50 BBHs. Phylogenetic reconstructions including HLS were performed from aa sequences with the same methods as above. The tree presented on Figure 8 was built from an alignment of length 165 aa, using a WAG+**Γ** model chosen with ProtTest [89]

### Data deposition

The complete OtV5 DNA sequence has been deposited in GenBank, accession number EU304328. New algal strains have been deposited in the Roscoff Culture Collection [15], France.

## Supporting Information

Table S1 Presence of Pfam motifs in putative OtV5 proteins. Pfam motifs were detected using the pfam_scan.pl script with default values. Motifs corresponding to the same CDS product are grouped and coloured alternatively red and blue. Amino acid sequences with no detectable motif are not presented. Columns show respectively the OtV5 gene name, start and end coordinates on the amino acid sequence, the Pfam accession number (linked to the corresponding Pfam web page), indication of complete (ls) or partial (fs) motifs, Bit value, e-value, Pfam ID and whether the motif is nested in another motif.
(0.19 MB XLS)

Table S2 Comparison of OtV5 with other algal viruses. OtV5: *O. tauri* virus strain 5; PBCV-1: *P. bursaria* chlorella virus strain 1; *P. bursaria* chlorella virus strain NY-2A; MT325: *P. bursaria* chlorella virus strain MT325; EhV-86: *E. huxleyi* virus strain 86. For each gene, numbers correspond to the number of copies of this gene family in the genome. Highlighting colors: yellow-common to characterized micro-algal large DNA viruses, orange-common to characterized phycodnaviruses of Chlorophyta, green-not present in other characterized phycodnaviruses.
(0.03 MB XLS)
